# Announcement: 6^2^ Iternational  Symposium on Metal Ions in Biology and Medicine

**DOI:** 10.1155/MBD.1999.63

**Published:** 1999


					ON
6th INTERNATIONAL SYMPOSIUM
METAL IONS IN BIOLOGY AND MEDICINE
7 10 May 2000
Caribe Hilton, San Juan, Puerto Rico, USA
DESCRIPTION:
The 6th International Symposium on Metal Ions in Biology and Medicine will be held in San
Juan, Puerto Rico, USA. The objective of this symposium is to foster exchange of opinions
between professionals and specialists working on analysis, research and applications of
metals, trace elements and minerals in biological, biochemical, medical sciences, and
environmental health. The scientific program, composed of plenary and concurrent sessions,
poster presentations, and panel discussions, is designed to promote intensive and productive
dialogue among experts in these fields. Short courses have been also organized, featuring
specialized areas including toxicology, analysis, and environmental health research. The
organizers of this symposium welcome you, and hope that you will enjoy the scientific and
social programs.
HISTORICAL PERSPECTIVE:
The International Symposium on Metal Ions in Biology and Medicine was originated in 1990 in
Reims, France, and it has been continued every two years. The chronological sequence of
previous conferences is listed below:
Location & Year
1st: Reims, France, 1990
2nd Loutraki, Greece, 1992
3rd: Montreal, Canada, 1994
4th: Barcelona, Spain, 1996
5th Munich, Germany,1998
Chairpersons
Dr. Ph. Collery and Dr. J.C. Etienne
Dr. J. Anastassaoupoulou, Dr. T. Theophanides
Dr. N.A. Littlefield and Dr. L. Poirier
Dr. J.L. Domingo, Dr. J.M. Llobet, Dr. J. Corbella
Dr. P. BrAtter, Dr. V. Negretti de Br&tter, Dr. P. Schramel
Members of the International Scientific Committee are named by the new organizers.
Former chairpersons remain permanently in the International Scientific Committee.
SCOPE OF THE SYMPOSIUM:
The scientific program is organized according to traditional areas in biology, medicine, and
environmental health. The following topics are examples of the intended breadth of the
Symposium:
Metals and Environmental Health
Speciation of Metals and other Elements
Uses of Metals in Clinical Applications
Epidemiology and Occupational Health
Metals and Disease: Environmental and Toxicologic Pathology
Health Effects of Arsenic
Metals and Aging
Metals and Homeostasis
Effects of Low and High Nutritional Trace Elements Intake
Risk Assessment of Trace Element Status and Health
Toxicity of Metals
Metals and Hormone Receptors
Metals and Chelation Therapy
Metals and Enzyme Activity
Advanced Methods for the Analysis of Trace Elements and Metal Ions
The objectives of the Symposium are"
To promote the exchange and dissemination of scientific knowledge about trace elements
and metal ions and their role in biological processes and in the etiology of diseases
including cardiovascular diseases, diabetes, and cancer.
63
2 To explore new advances, applications and uses of metal ions on medical research areas
including: clinical applications, aging, nutrition, and cancer prevention.
3 To discuss new technological developments on the analysis of trace elements.and metal
ions.
4 To foster discussion on environmental and public health research needs.
INTEREST GROUPS:
The Symposium is intended to bring together scientists and specialists from the following
areas:
Medical scientists, health professionals, toxicologists, pharmacologists; and others interested
in the effects of metal ions, trace elements, and minerals on human and environmental health.
Environmental and industrial scientists, epidemiologists, geo-scientists.
*Biochemists, biologists, chemists.
Microbiologists, geneticists, molecular biologists, cell biologists.
COMMITTEES:
Chairman, International Scientific Committee:
Dr. Jos A. Centeno
Dept. of Environmental and Toxicologic Pathology, Armed Forces Institute of
Pathology, Washington, DC 20306-6000
Phone: (202) 782-2839; Fax: (202) 782-9215; Email: centeno@afip.osd.mil
Co-Chairman: Dr. Philippe Collery (France), Institut International de Recherche sur les Ions
Mtalliques, Email: Philippe.collerv@wanadoo.fr
Honorary Chairpersons:
Dr. Florabel G. Mullick (USA), Center for Advanced Pathology, AFIP, Email:
Mullick@afip.osd.mil
Prof. Dr. Jean-Claude Etienne (France), Conseil Rgional de Champagne Ardenne,
http://www.iirim.com
General Secretary: Dr. Lylia Khassanova (Russia), http://www.iirim.com
International Scientific Committee:
C. Abernathy (USA)
O. Andersen (Denmark)
G. Beckett (Scotland, UK)
P. Br&tter (Germany)
M. Cebrian (Mexico)
J. Corbella (Spain)
M. Costa (USA)
F.A. de Wolff (The Netherlands)
J. Durlach (France)
M.F. Flores-Arce (Mexico)
M. Gielen (Belgium)
K. Irgolic (Austria)
K.S. Kasprzak (USA)
J.M. Llobet (Spain)
B. Michalke (Germany)
J. Neve (Belgium)
E.A.R. Oiler (USA)
L.A. Poirier (USA)
S.K. Shukla (Italy)
F.W. Sunderman, Jr. (USA)
F. Tchernitchin (Chile)
C. Thompson (USA)
G. Vernet (France)
B.S. Zheng (China)
J. Anastassopoulou (Greece)
V.H. Aposhian, (USA)
A. Berthelot (France)
S. Caroli (Italy)
B. Conard (Canada)
R. Cornelis (Belgium)
R. Deloncle (France)
J.L. Domingo (Spain)
B. Farzami (Iran)
B. Fowler (USA)
O. Guillard (France)
V. Kalfakakou (Greece)
N.A. Littlefield (USA)
D.N.G. Mazumder (India)
V. Negretti de Br&tter (Germany)
G. Nowak (Poland)
A. Pineau (France)
P. Schramel (Germany)
M. Simonoff (France)
K.T. Suzuki (Japan)
Th. Thophanides (Greece)
D. Templeton (Canada)
M.P. Waalkes (USA)
64
Organizing Committee:
Dr. Herman Gibb, Co-Chairman,
U.S. Environmental Protection Agency; Email: g.ibb.herman@epamail.epa.gov
Dr. Michalann Harthill, Co-Chairman
U.S. Geological Survey; Email: michalann harthill@usgs.gov
Local Organizing Committee:
Dr. Jaime Matta, Co-Chairman
Ponce School of Medicine, Puerto Rico; Email: 103650.1713@compuserv.com
Dr. Jorge I. Vlez Arocho, Co-Chairman
Center for Hemispherical Cooperation in Science and Engineering
University of Puerto Rico-Mayageez; Email: cohemis@exodo.upr.clu.edu
JOINTLY SPONSORED BY:
American Registry of Pathology (ARP)
Armed Forces Institute of Pathology (AFIP)
Institut International de Recherche sur les Ions Mtalliques (llRIM-Reims,France)
University of Puerto Rico at Mayageez (UPRM)
U.S.-Environmental Protection Agency (US-EPA)
U.So-Geological Survey (US-GS)
National Cancer Institute (NCI)
National Institute of Environmental Health Sciences (NIEHS)
Conseil Rgional de Champagne Ardenne (CRCA-Reims, France)
Ana G. Mendez University System of Puerto Rico (AGMUS)
UNDER THE AUSPICES OF:
Federation of European Societies on Trace Elements and Minerals (FESTEM)
Center for Hemispherical Cooperation on Science and Engineering, UPRM
Ponce School of Medicine, Puerto Rico (PSM-PR)
Societe pour le Developpment des Recherches sur le Magnesium (SPDRM)
International Association of Geochemistry and Coscochemistry (IAGC)
SYMPOSIUM SERVICE OFFICE:
American Registry of Pathology
Department of Medical Education, Armed Forces Institute of Pathology
Washington, DC 20306-6000
Toll Free Tel: (800) 577-3749 (U.S. only)
Tel: (202) 782-2637 Fax: (202) 782-5020
International Toll Free Fax: (877) 891-3482
Web Site: http://www.afip.org
Hotel Accomodations:
Registration Affairs:
Exhibits and Sponsorships:
Mrs. Lisa Holmes; Email: Holmes@afi).osd.mi!
Mr. Ontee Biggs; Email: biggs@, afip.osd.mil
Ms. Rene Sutton; Email: .utton..@afip.osd.mil
Language: English
Registration Fees*:
$400 full participants
$250 one day participants (coffee-breaks included)
$285 students and retired scientists (welcome reception, coffee breaks included)
$185 accompanying person (welcome reception, coffee-breaks and symposium banquet
dinner included)
*Registration fees include access to lecture hall, Book of Abstracts, Book of Proceedings,
welcome reception, symposium banquet dinner, and coffee-breaks.
Proceedings: The symposium proceedings will be published by John Libbey Eurotext
Publishers (Gilles Cahn, Editor-in-Chief) and indexed in the principal
international databases, including Current Contents.
SECOND ANNOUNCEMENT AND CALL FOR PAPERS:
The 2nd Symposium Announcement, with registration card and hotel information, will
be published in May-June 1999. In addition, the 2nd Announcement will contain further
information about the Call for Abstracts, registration, the scientific program, travel
arrangements, accommodations, and social events. Social events include a Welcome
Reception Sunday evening and the Symposium Banquet Dinner for all registrants and their
accompanying guests; scheduled tours will be available at cost to historic attractions in Old San
Juan, the National Rain Forest "El Yunque", the Ponce Museum of Arts, and many other sites.
Please contact the Symposium Service Office before May 1999 for subsequent mailing.
6th International Symposium on Metal Ions in Biology and Medicine
Symposium Service Office
Department of Medical Education
Attention" Dr. Jos A. Centeno, Chairperson
American Registry of Pathology
Armed Forces Institute of Pathology
Washington, DC 20306-6000
E-mail: centeno@afip.osd.mil

				

